# Pandemic-response adenoviral vector and RNA vaccine manufacturing

**DOI:** 10.1038/s41541-022-00447-3

**Published:** 2022-03-02

**Authors:** Zoltán Kis, Kyungjae Tak, Dauda Ibrahim, Maria M. Papathanasiou, Benoît Chachuat, Nilay Shah, Cleo Kontoravdi

**Affiliations:** 1 The Sargent Centre for Process Systems Engineering, Department of Chemical Engineering, Imperial College London, South Kensington Campus, London, SW7 2AZ UK; 2 Department of Chemical and Biological Engineering, The University of Sheffield, Mappin Street, Sheffield, S1 3JD UK

**Keywords:** Biotechnology, RNA vaccines, DNA vaccines, Viral infection

## Abstract

Rapid global COVID-19 pandemic response by mass vaccination is currently limited by the rate of vaccine manufacturing. This study presents a techno-economic feasibility assessment and comparison of three vaccine production platform technologies deployed during the COVID-19 pandemic: (1) adenovirus-vectored (AVV) vaccines, (2) messenger RNA (mRNA) vaccines, and (3) the newer self-amplifying RNA (saRNA) vaccines. Besides assessing the baseline performance of the production process, impact of key design and operational uncertainties on the productivity and cost performance of these vaccine platforms is quantified using variance-based global sensitivity analysis. Cost and resource requirement projections are computed for manufacturing multi-billion vaccine doses for covering the current global demand shortage and for providing annual booster immunisations. The model-based assessment provides key insights to policymakers and vaccine manufacturers for risk analysis, asset utilisation, directions for future technology improvements and future epidemic/pandemic preparedness, given the disease-agnostic nature of these vaccine production platforms.

## Introduction

The COVID-19 pandemic, caused by the SARS-CoV-2 virus, created an unprecedented demand for rapid, large-scale vaccine deployment that the world is struggling to meet. This urgency and scale of immunisation against a new disease poses enormous challenges on the entire vaccine deployment pipeline^1–4^. This pipeline has the following main parts: (1) pre-clinical development and testing, (2) clinical development and testing, (3) production process development, scale-up and technology transfer for the manufacturing of the vaccine active ingredient (drug substance, DS), (4) sourcing of raw materials and consumables for manufacturing both the DS and the final packaged vaccine product filled into glass vials or other containers (fill-to-finish processes), (5) DS production under current Good Manufacturing Practices (cGMP), (6) fill-to-finish processes under cGMP, (7) vaccine distribution and (8) vaccine administration to the population^1–4^.

Global COVID-19 vaccination programmes have been constrained by vaccine manufacturing capacity, particularly in low- and middle- income countries^5,6^. In response to this, vaccine manufacturing for pandemic-response production started “at risk”, before the completion of clinical trials^4,7^ and before the development, optimisation and scale up of production processes^1^, leading to large uncertainty in the DS amount per dose and number of doses per person. Tackling future virus variants will furthermore require new vaccine designs, while manufacturing needs to be low-cost to enable rapid mass immunisation worldwide^1,8^.

The focus of this paper is on the manufacturing processes for adenovirus-vectored (AVV), messenger RNA (mRNA), and the newer self-amplifying RNA (saRNA) vaccines. These vaccines contain genetic instructions, in the form of DNA for the AVV vaccine and RNA in case of the mRNA and saRNA vaccines, based on which the cells of the human body produce the vaccine antigen, such as the spike protein of the SARS-CoV-2 virus^9–14^. Because only the genetic instruction and not the antigen is produced, the vaccine production processes serve as platform technologies. A platform technology implies that once validated and established at production scale, the same production process can produce a wide range of different vaccines and vaccine candidates against both known and future pathogens. The AVV and mRNA vaccine platforms have matured in terms of technology development and have reached a technology readiness level (TRL) of 8 C or higher^15^. On the other hand, the saRNA vaccine platform is currently in clinical development (TRL between 6 C and 7B)^15^, as currently no saRNA vaccine has demonstrated efficacy against SARS-CoV-2 or any other disease. To enable rapid vaccine development it is also crucial to enable fast antigen identification and design, ideally by using prioritisation and prior knowledge from prototype pathogens^16,17^. For this, antigenic target knowledge, virology knowledge, assays for pre-clinical and clinical development, animal models and other learnings can be transferred to develop vaccines against similar pathogens, for example from the same family, genus, species or group^16,17^.

Herein, key uncertainties and their impact on COVID-19 vaccine production are analysed and the production process scales, timescale and manufacturing resources required for producing 1 billion COVID-19 vaccines per year are estimated. These estimates can serve as a basis for calculating the requirements to produce vaccines for global demand. Three fill-to-finish technologies are also evaluated with respect to their pandemic-response manufacturing performance: conventional fill-to-finish in 5-dose or 10-dose vials, blow-fill-seal in single-dose vials, and the new 200-dose bag Intact™ Modular Filler^1,18–20^.

The mathematical models used to conduct the assessment are representative of industrial COVID-19 vaccine manufacturing processes and compliant with cGMP regulations^21,22^. Because the relevant technologies and their productivity (number of doses produced per unit time and unit scale) vary enormously across the three vaccines, two key performance indicators (KPIs) are used for their comparison on an equal basis: annual production (doses per year), and cost per dose (USD per dose). The effect of key model input uncertainties on these KPIs are also quantified using global sensitivity analysis^23–25^. Finally, the batch production rates are estimated for each technology, as the shorter these are, the faster vaccines can be made available for administration. The results of this study can inform policy makers and vaccine manufacturers on how to improve manufacturing and asset utilisation against COVID-19 and its variants, but also against future outbreaks due to the disease-agnostic nature of these vaccine production platforms^1,26^.

## Results and discussion

The AVV, mRNA and saRNA DS production processes (primary manufacturing) and fill-finish processes are described in Supplementary Section 1.1–1.4 of the Supplementary Information document—see also Section “Methods”. The uncertain input factors for the three platforms and corresponding ranges of variation are listed in Table 1. A triangular probability distribution was assumed for those input factors with a highly-probable central value, and a uniform probability distribution for those having a similar probability over their variation range or not being well understood. Refer to Supplementary Section 2.2 for further information on data sources for sensitivity analysis, assumptions, justification for the uncertainty ranges, and factors influencing these ranges.

**Fig. 1:**
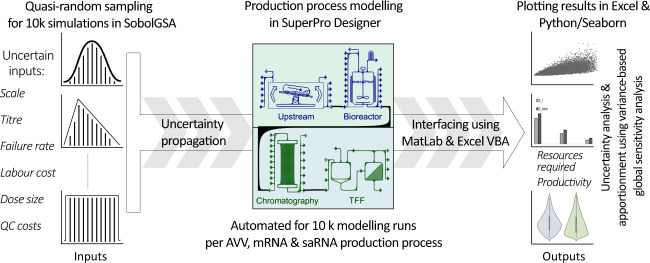
Computational framework for uncertainty quantification in vaccine production platforms. The aim of this approach is to evaluate process performance under uncertainty and variability resulting from both the design and operation of the new vaccine production platform technologies. The uncertainty is propagated from input factors to key performance indicators (KPIs) via the mathematical model. Then, the KPI variation ranges are apportioned back to each input factor as sensitivity indices. Input factors include scale of production process, batch failure rate, titre/yield in the production bioreactor, cost of labour, drug substance amount per dose, and cost of quality control. KPIs include capital investment cost requirements, operating costs, number of batches produced per year, amount of drug substance produced per batch, amount of drug substance produced per year, number of doses produced per batch, number of doses produced per year, and production cost per dose.

A variance-based global sensitivity analysis^27–29^ was conducted to quantify how uncertain input factors propagate to KPIs, then aportion the resulting KPIs variation ranges back to each input factor as sensitivity indices to reveal any synergetic/antagonistic effects^27–29^ (cf. Fig. 1). Sensitivity and uncertainty analyses were not performed for the fill-to-finish processes as these are well-established technologies (relevant values are shown in Supplementary Table 1).

### Comparative techno-economic assessment of COVID-19 vaccine production platforms

**Fig. 2:**
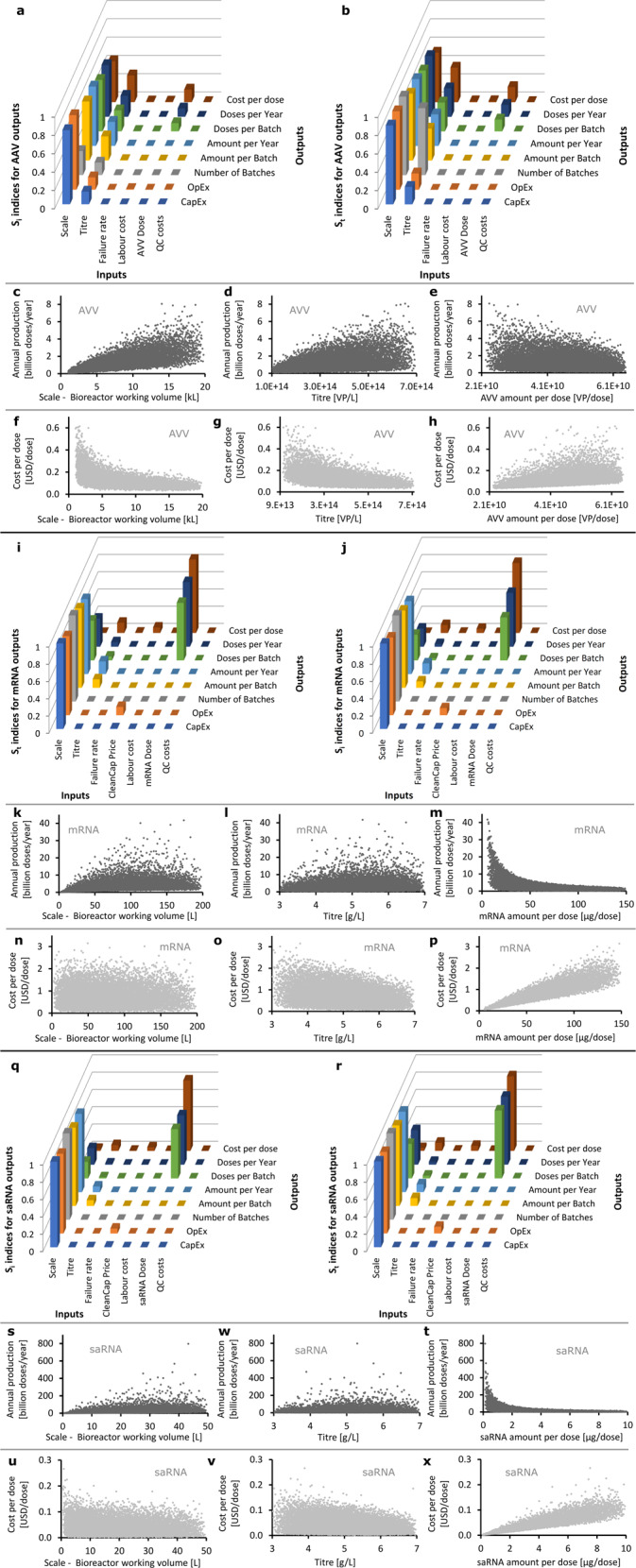
Global sensitivity analysis of multiple input factors for AVV, mRNA and saRNA platforms on key performance indicators (KPIs)^27–29^. The input factors are scale, titre, failure rate, CleanCap purchase price (for mRNA and saRNA only), labour cost, drug substance (AVV or RNA) amount per dose, and quality control (QC) cost. The KPIs are capital costs (CapEx), operating costs (OpEx), number of batches produced per year, amount per batch, amount per year, number of doses per batch, number of doses per year (annual production amounts), and cost per dose. **a**–**h**, **i**–**p**, **q**–**x**: vaccine drug substance production performance for AVV, mRNA and saRNA platforms. **a**, **b**, **i**, **j**, **q**, **r**: first-order effect (S_i_) and total effect (S_t_) sensitivity indices for the KPIs versus each input factor for the AVV, mRNA and saRNA platforms. Large S_i_ and S_t_ values indicate a strong impact of given input factor (*X*-axis) on KPI (*Y*-axis), while low S_i_ and S_t_ values indicate a weaker dependence of the KPI on the input factor. **c**–**h**, **k**–**p**, **s**–**x**: magnitude of the random co-variation of AVV, mRNA and saRNA drug substance annual production amounts, and cost per dose against production scale, titre, and drug substance amount per dose. Dots clustered around a narrower band indicate that the given input factor explains most of the KPI variance, while dots spread out over a wider band suggest that the input parameter explains little or none of the KPI variance.

Sensitivity analysis results for each of the platform processes are shown in Fig. 2. The bar charts show the impact of input factors (6 for AVV and 7 for mRNA and saRNA) onto 8 KPIs, while the scatter plots show the impact of each input factor onto each KPI. In the AVV case, the annual production amount (cf. doses per year) is mostly influenced by scale, followed by titre, and then AVV amount per dose (cf. Fig. 2a, b). The magnitude by which these three key factors impact the annual production amount are captured in Fig. 2c–e. Likewise, the AVV cost per dose is mostly impacted by uncertainty in scale, titre, and AVV amount per dose (cf. Fig. 2a, b, magnitude shown in Fig. 2f–h). Overall, the production scale followed by titre has the largest impact on all 8 outputs for the AVV process (cf. Fig. 2a, b).

In both mRNA and saRNA cases, the annual production amount depends mostly on the RNA amount per dose, followed by the production scale (cf. Fig. 2i–m for mRNA and Fig. 2q–t for saRNA). The RNA amount per dose is also dominent on the cost per dose, followed to a lesser extent by the production titre and the price of the CleanCap 5' cap analogue (cf. Fig. 2i, j, n–p for mRNA, Fig. 2i, j, u–x for saRNA, and Supplementary Fig. 2 for the effect of CleanCap 5' cap analogue purchase price on the cost per dose). Overall, the RNA amount per dose has the highest impact on the cost per dose, doses produced per year, and on doses produced per batch, whereas the production scale has the highest impact on the remaining five KPIs (cf. Fig. 2i, j, q, r). The small difference in magnitude between the first-order effect (S_i_) and total effect (S_t_) sensitivity indices indicates that all input factors have predominantly separable (additive) effects on the KPIs, that is, interactions between multiple factors on the KPIs are minor.

The sensitivity analysis reveals that process scale is the key lever for increasing the AVV vaccine annual production amount and reducing its cost per dose, followed by the production titre and the AVV amount per dose. Scaling up the AVV production process would further increase the already high capital expenditures (cf. Supplementary Fig. 1 and Supplementary Table 2), and keeping such large-scale production processes idle or under-utilised (e.g. for surge capacity in non-pandemic times) could incur high fixed costs as well. This is because AVV production costs are dominated by fixed costs, such as facility-dependent costs and labour costs (cf. Supplementary Fig. 3), which is typical of cell-based vaccine and biopharmaceutical processes. The AVV production titre can be increased either by culturing the host (e.g. HEK293) cells at higher densities, or by achieving higher specific productivity (viral particles per cell per unit time). The former may be achieved by process intensification and the latter via genetic engineering adenoviruses or host production cells. The AVV amount per vaccine dose depends on the potency of the expressed antigen and the efficiency with which AVVs infect human cell and induce antigen expression.

The main driver to increasing the RNA vaccine annual production amount and reducing the cost per dose is by decreasing the RNA amount per dose and, to a lesser extent, by increasing the production scale and titre. In principle, a reduction in the RNA amount per dose may be achieved by using saRNA or by improving the RNA delivery efficiency or antigen expression levels from the RNA. The potency of the antigen can also impact the amount of RNA that is needed per vaccine dose. The scale of the RNA production process can be further increased, as RNA vaccine production has a relatively low facility footprint (cf. Supplementary Fig. 1 and Supplementary Table 2)^8,30,31^. RNA vaccine production has relatively low fixed costs and high variable costs (cf. proportion of variable costs such as material and consumable costs in Supplementary Fig. 3). Unlike the AVV production process, the RNA platform technology would therefore be suitable for maintaining surge capacity from a cost perspective as idling or under-utilising RNA vaccine production processes would not incur high fixed costs. The RNA vaccine production titre could be enhanced by protein engineering of more productive RNA synthesising enzymes, by process intensification, or by operating the production process in fed-batch or continuous mode^32,33^.

Next, the AVV, mRNA and saRNA platform technologies are compared in terms of their productivity, by considering DS production only and assuming a unique production line at a single facility. This comparison furthermore assumes production processes that are fully developed, validated and implemented at production scale. Because the complexities and times required for setting up production may differ for the three platform technologies, these set-up, validation and start-up times were not accounted for either—refer to Section “What resources and capacity for producing multi-billion doses of Covid-19 vaccine?” regarding the costs associated with setting up production based on these platform technologies and those related to operating these production lines and facilities.

**Fig. 3:**
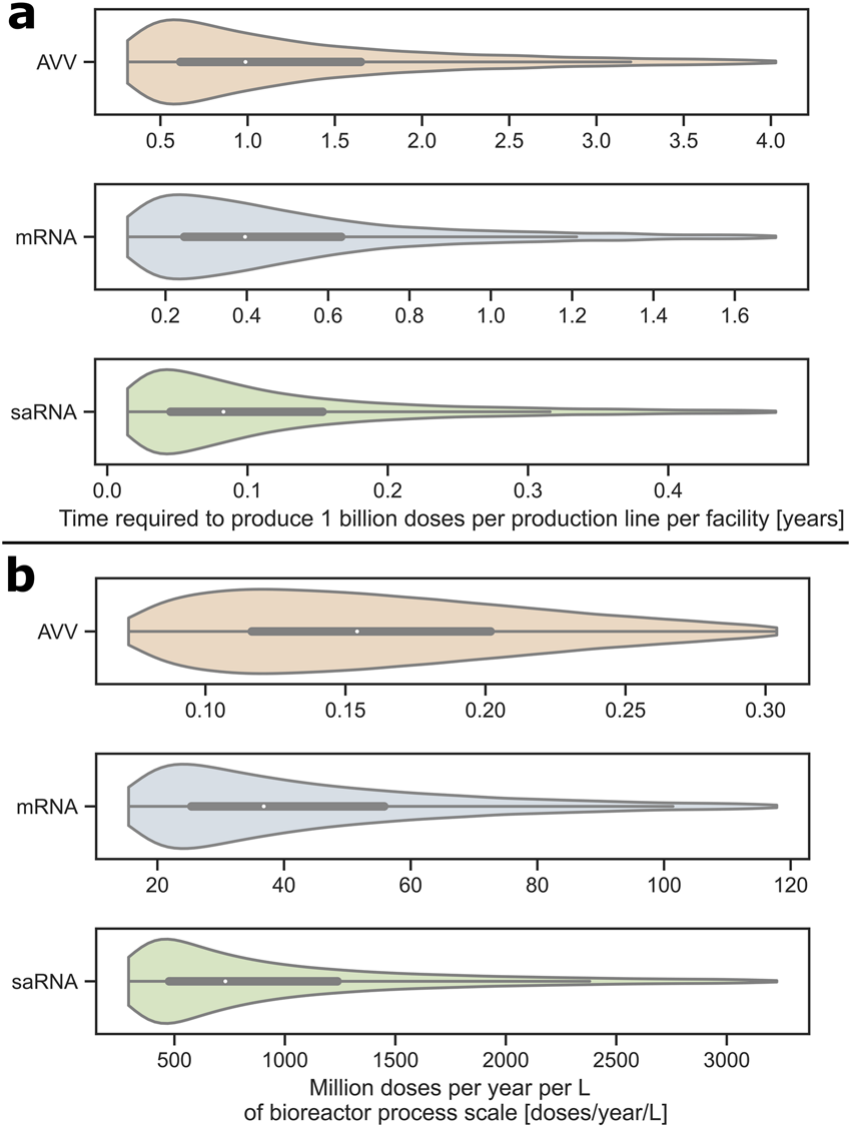
Comparison of the AVV, mRNA and saRNA vaccine production platforms under the uncertainty scenarios in Table 1. **a** Violin plots of the required times for producing 1 billion doses of AVV, mRNA and saRNA vaccine drug substance. **b** Violin plots of the number of vaccine doses produced per year and unit bioreactor working volume. The box and whiskers inscribed within each violin plot depict the interquartile range and full percentile range (excluding outliers), respectively, and the median value is indicated by the white dot inside each box. The bottom 5% and top 5% of all values were excluded from all violin plots to enable a better visualisation of the region of interest around the box plot—cf. Supplementary Fig. 4 for violin plot comparisons on the same *x*-axis and including the full data range.

The manufacturing times needed for producing 1 billion doses of DS (excluding quality control testing) with each technology and the corresponding productivities are compared in Fig. 3. In addition to reporting the mean and interquartile ranges, the violin plots show the full distribution shape of the KPIs. Note that the *x*-axis ranges are different in each plot of Fig. 3 to illustrate the differences in distribution shape—the same violin plots are compared in Supplementary Fig. 4 to illustrate the differences in magnitude across platforms.

In a facility with a single production line (cf. Fig. 3a), the AVV platform is predicted to require by far the longest time for producing 1 billion vaccine doses of DS (median (M) = 0.99 year (yr), interquartile range (IQR) = 0.58–1.77 yr). The saRNA platform is predicted to be the fastest (M = 0.083 yr, IQR = 0.042–0.166 yr), followed by the mRNA platform (M = 0.40 yr, IQR = 0.23–0.67 yr). But notice that the mRNA platform, or even the AVV platform, could outperform the saRNA platform under certain uncertainty realisations given the overlap between the probability distributions in Fig. 3a. Nevertheless, the right skew of these probability distributions for all three platforms indicates that the time required to produce 1 billion doses per production line is likely to be at the lower end of the scale and, only in very rare scenarios, would this production time be at the higher end of the scale.

The AVV platform relies on cell-based production, which introduces more biological variability and is more prone to failure compared to the mRNA and saRNA platforms. This was taken into account through a uniform failure-rate distribution between 0 and 15% for AVV production and 0–10% for mRNA and saRNA production. To capture the difference in production scale across the three vaccine platforms, another comparison in terms of their specific productivity (expressed in million doses produced per year and unit bioreactor working volume) is presented in Fig. 3b. The saRNA platform (M = 730, IQR = 456–1332 × 10^6^ doses L^−1^ yr^−1^) is predicted to be 20 times more productive than the mRNA platform (M = 36, IQR = 24–60 × 10^6^ L^−1^ yr ^−1^), which is itself 200–300 times more productive than the AVV platform (M = 154, IQR = 112–208 × 10^3^ L^−1^ yr^−1^). This large productivity difference between RNA and AVV platforms is due to the highly concentrated, cell-free nature of RNA production and its considerably shorter batch cycle times. The productivity difference between mRNA and saRNA vaccines is due to the substantially lower amount of RNA per dose of saRNA vaccine. The right skew of the probability distributions in Fig. 3b furthermore suggests that the realised productivities of all three platforms might be significantly lower than the median productivity values.

Fill-to-finish is initially delayed by the time required to produce and perform the QC test of the first DS batch. During pandemic-response manufacturing, the DS may be produced and stockpiled while awaiting the clinical trial results^34^, in which case fill-to-finish would be further delayed relative to the start of production campaign. Bottlenecks in the DS manufacturing depend on the specific vaccine platform technology. For AVV, the bottleneck is caused by the time needed by the mammalian cell culture to reach sufficient amount for the production bioreactor. For mRNA and saRNA, the bottleneck lies in the LNP formulation operations. The equipment used for the LNP formulation can be microfluidics^35,36^, impingement jet mixers^31^, T-junction mixer^36^, multi-inlet vortex mixers^37^ or pressurised tanks^30^. Herein, the LNP unit operation was modelled based on times required for parallelised microfluidics LNP formulation devices and the cost of the four lipids was estimated. But substantial additional license fees might be payable for the ionisable lipid. This bottleneck may be removed by increasing the size (scale-up) or the number (scale-out) of parallel equipment for the formulation unit operation. In addition, mRNA vaccine production is most effectively enhanced by reducing the mRNA amount per vaccine dose, given the multiplicative-inverse relationship between annual production amount and amount per dose (cf. Supplementary Fig. 1H and Fig. 2m).

On top of these production bottlenecks, further lead times may be expected for the completion of certain QC tests, especially with new platform technologies such as RNA vaccines. Deployment of a Quality-by-Design (QbD) framework could help streamline vaccine manufacturing by building quality assurance into the design and operation of the production processes, which is currently limited by lack of suitable process analytical technology (PAT)^38^.

Importanly, fill-to-finish technologies (secondary manufacturing, cf. Supplementary Section 1.4 and Supplementary Table 1)^1,18–20^ may also shift the production bottleneck. In combining a single AVV DS production line with a single 10-dose vial filling line (400 doses min-1 at 60% overall equipment effectiveness), the bottleneck is in the DS production under the baseline scenario (2000 L bioreactor working volume scale, cf. Supplementary Table 2. But the bottleneck shifts to fill-to-finish for larger DS production scales (compare values in Supplementary Table 1 and Fig. 3a). Likewise, the baseline mRNA DS production rate (cf. Supplementary Table 2) is slower than the filling rate into 5-dose vials, or even into 10-dose vials (400 vials min^−1^, e.g., Moderna vaccine). Lastly, combining one saRNA DS vaccine production line with a single fill-to-finish line into 5-dose vials (400 vials min^−1^) would shift the production bottleneck to the fill-to-finish stage. This saRNA fill-to-finish bottleneck could in principle be removed in the future by using technologies such as the new 200-dose bag Intact™ Modular Filler^1,18–20^.

### What resources and capacity for producing multi-billion doses of Covid-19 vaccine?

**Fig. 4:**
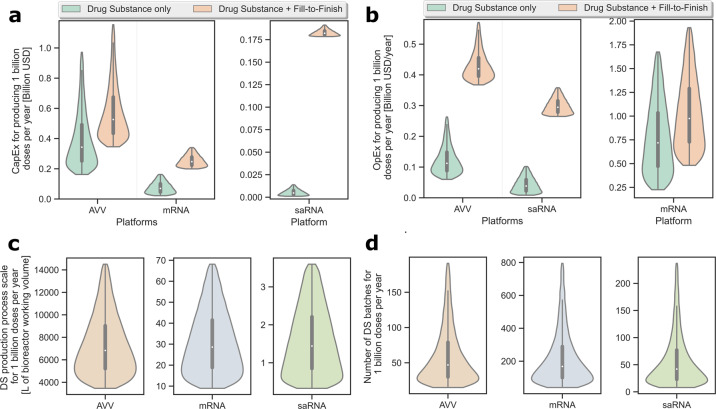
Projection of the resources and capacity needed to produce 1 billion COVID-19 vaccine doses per year using the AVV, mRNA and saRNA platform technologies. The analysis considers the same uncertainty scenarios as in Table 1, and assumes AVV vaccines filled into 10-dose vials and mRNA/saRNA vaccines filled into 5-dose vials. **a** Violin plots of the capital costs (CapEx), both without and with fill-to-finish. **b** Violin plots of the operating costs (OpEx), both without and with fill-to-finish. **c** Violin plots of the required production process scales, expressed per unit bioreactor working volume. **d** Violin plots of the required numbers of batches. The box and whiskers inscribed within each violin plot depict the interquartile range and full percentile range (excluding outliers), respectively, and the median value is indicated by the white dot inside each box. The bottom 5% and top 5% of all values were excluded from all violin plots to enable a better visualisation of the region of interest around the box plot—cf. Supplementary Fig. 5 for violin plot comparisons on the same *y*-axis and including the full data range.

One may extrapolate the model-based assessment and uncertainty quantification conducted in Section “Comparative techno-economic assessment of COVID-19 vaccine production platforms” to predict the capacity and resources needed for producing 1 billion doses of vaccine DS per year (cf. Fig. 4). A linear extrapolation of the AVV, mRNA and saRNA production processes is relevant insofar as the simulations already describe large-scale processes for making multi-billion doses of vaccine DS. Economy of scale therefore plays a lesser role in scaling up or scaling out such processes and is counteracted by likely difficulties for suppliers to meet such high demands in raw materials^39–42^. It is estimated that 11.3 billion COVID-19 vaccine doses are required globally to reach herd immunity^6^. Predictions of the costs and capacity required to meet this global demand with each technology could be further extrapolated from Fig. 4.

The CapEx and OpEx predictions in Fig. 4a, b regarding the production of 1 billion vaccine doses per year consider the same uncertainty scenarios as in Table 1. They furthermore assume AVV vaccines filled into 10-dose vials (cf. Oxford/Astrazeneca) and mRNA/saRNA vaccines filled into 5-dose vials (cf. BioNTech/Pfizer). The corresponding production scales and overall number of production batches are shown in Fig. 4c, d. It is worth noting that all probability distributions in Fig. 4 display a right skew, meaning that scenarios below the median are more likely to materialise—the same comparison is presented in Supplementary Fig. 5 on the same *y*-axis to illustrate the differences in magnitude across platforms.

**Table 1:** Input factors in global sensitivity analysis of mRNA, saRNA and AVV vaccine drug substance production.

Parameter name and unit	AVV		mRNA and saRNA (RNA)		Uncertainty distribution^a^	Ref.
	Range	Central value^b^	Range	Probable value		
Process scale [L for RNA and AVV]	1000–20,000	2000	2–200 mRNA 0.5–50 saRNA	30 mRNA 5 saRNA	Triangular	^30,31,45,46,71,72,110^
Process failure rate [%]	0–15	8	0–10	5	Uniform	^111,112^
Production titres [g L^−1^ for RNA; viruses L^−1^ for AVV]^c^	1 × 10^14^ –7 × 10^14^	2.5 × 10^14^	3–7	5	Triangular	^32,45,46,63–73,85–88^
5' cap analogue cost [USD g^−1^]	N.A.	N.A.	2500–10,000	3000	Triangular	cf. SI
Basic labour rate [USD h^−^^1^]	5–30	23	5–30	23	Triangular	^111,113–115^
Drug substance amount per dose [µg dose^−1^ for RNA; viruses dose^−1^ for AVV]	2.2 × 10^10^ – 6.5 × 10^10^	5 × 10^10^	5–150 mRNA 0.1–10 saRNA	30 mRNA 1 saRNA	Triangular	^67,79,80,89–92,116–121^
Cost of Lab/QC/QA [% of total labour costs]	30–80	60	30–80	60	Uniform	^111,122^

^a^Uncertainty distribution assumed either triangular (with highest probability for the central value) or uniform.

^b^Central values corresponding to the baseline scenarios (cf. Table S2). For a triangular distribution the central value is also the mode. For a uniform distribution the central value has the same probability as the other values in range. The superscript “−1” means power of “−1”, which is mathematical notation for multiplying by the inverse, which is equivalent to division.

^c^Combined variations of product titres in the bioreactor and recovery losses in downstream purification.

Of the three technologies, the AVV platform is predicted to have the highest CapEx (M = 343, IQR = 243–516 million USD) to produce 1 billion vaccine DS doses per year. The AVV platform also requires the largest production scale (M = 6828, IQR = 5061–9365 L bioreactor working volume), and second highest OpEx (M = 112, IQR = 85–154 million USD yr^−1^). Since the AVV platform is commonly deplayed at scales of 2000 L bioreactor working volume or above, it requires a low number of batches to meet the production target (M = 47, IQR = 28–84 batches yr^−1^).

The mRNA platform requires the highest number of batches (M = 168, IQR = 93–310 batches yr^−1^), highest OpEx (M = 720, IQR = 446–1076 million USD yr^−1^), second highest CapEx (M = 70, IQR = 44–105 million USD) and second highest production scale (M = 29, IQR = 18–43 L bioreactor working volume). These cost and productivity KPIs are based on 30 µg mRNA per dose (cf. BioNTech/Pfizer) and would thus be less favourable for a higher mRNA amount per dose (cf. Moderna, 100 µg mRNA per dose), given the inverse proportional relationship between annual mRNA DS production and amount per dose (cf. Supplementary Fig. 1H and Fig. 2m).

An saRNA platform would require both the lowest CapEx (M = 5, IQR = 3–8 million USD) and lowest OpEx (M = 38, IQR = 21–62 million USD yr^−1^). It would furthermore require the lowest production scale (M = 1.44, IQR = 0.79–2.31 L bioreactor working volume) and, depending on the uncertainty realisation, the smallest number of batches (M = 42, IQR = 21–83 batches yr^−1^) as well.

The OpEx of the mRNA and saRNA vaccines is driven by the high material costs, due to the novelty and limited supply of some of the specialised raw materials. These include the 5' capping reagents (e.g. 5' capping analogues such as CleanCap and 5' capping enzymes), modified nucleotides (e.g. pseudouridine triphosphate used for the manufacture of Moderna and Pfizer/BioNTech Covid-19 vaccine), cationic lipids used in the LNP formulations, plasmid DNA and T7 RNA polymerase enzymes^8,43^. Moderna’s COVID-19 vaccine production process uses post-transcriptional enzymatic capping^44^, which requires high-cost capping enzymes (e.g. Vaccinia Virus Capping Enzyme in combination with 2'-O-methyltransferase enzyme) instead of the 5' capping analogues^45,46^. All of these raw material costs are expected to decrease over time as technologies and supply chains mature.

The production cost per dose, including fill-to-finish, is 0.54 USD for AVV vaccine in 10-dose vials and 2.39 and 0.39 USD for mRNA and saRNA vaccine, respectively, in 5-dose vials (cf. Supplementary Table 1 and Supplementary Table 2). Fill-to-finish is the dominant cost for saRNA vaccine (cf. Fig. 4a, b), due to the small DS amount per dose (0.1–10 µg dose^−1^, cf. Table 1). But it is comparable with the DS production cost for AVV vaccine, and even small compared to the DS production cost for mRNA vaccine due to the high variable costs of mRNA DS production (cf. Supplementary Fig. 3).

In principle, the DS amount per vaccine dose could be reduced, not only for mRNA vaccines but also AVV vaccines, which would improve manufacturability of second and third generation vaccines. This could be achieved by (i) designing more effective mechanisms to deliver the RNA or DNA payload into the cells, by improving the synthetic (e.g. LNP) and viral vectors, respectively, or (ii) enhancing antigen expression levels from the RNA or DNA for example by codon optimisation and by designing more effective untranslated regions on these genetic constructs^47,48^. In addition, the DS amount per dose can be antigen specific so antigen design may also play a crucial role (e.g. by stabilising the protein conformation)^47,49,50^.

The cost of establishing new production capacity based on any of the three platform technologies outweighs pademic-associated costs by several orders of magnitude when considering the mortality, healthcare burden, and economic recession caused by the COVID-19 pandemic. In particular, the pandemic cost on the global economy has been estimated at over 10 trillion USD^51,52^. These deleterious impacts can be mitigated by comparatively small investments to cover the capital and operating costs of vaccine manufacturing, as well as complementary investments for pathogen/antigen-specific research and development, pre-clinical and clinical development, distribution, storage, and administration of vaccines^53^. Ideally, such investments should be made in anticipation of a pandemic, considering the timescale required to build such manufacturing capacity over several years^1^. Manufacturing capacity based on platform technologies such as RNA and AVV could also be used for producing a wide range of vaccines over their lifetime. Besides financial resources, key raw materials (e.g. 5' cap analogues or cappin enzymes, cationic lipids and pseudouridine triphosphate), expertise and facilities are also in limited supply. Refer to^43^ for an analysis of the material, consumables, labour and facility requirements for mass-producing mRNA vaccines for pandemic response.

A large share of the COVID-19 vaccine shortfall is likely to be met by adapting or re-purposing manufacturing facilities used to manufacture other vaccines and biopharmaceuticals pre-pandemic. However, the healthcare impact of not sustaining routine childhood immunisations could quickly outweigh that of the COVID-19 pandemic, especially in Africa^54,55^. It is crucial, therefore, to manufacture and supply lifesaving vaccines against all vaccine-preventable diseases and to minimise the disruption in manufacturing of non-COVID-19 vaccines. This is even more important in the likely scenario of needing seasonal COVID-19 booster doses to immunise adults at risk of severe COVID-19 and frontline workers in the foreseeable future^1,26,56^. The global population that is vulnerable to COVID-19, including people aged over 60 and adults with underlying medical conditions, adds up to around 2.2 billion^57^. With an additional 200 million frontline personnel needding to also be immunised for preventing the spread of the disease^57^, an estimated 2.4 billion COVID-19 vaccine doses would be required annually in the single-dose booster scenario. The resource required to produce this many annual booster vaccinations using either of the three platform technologies are illustrated in Supplementary Fig. 6.

By investing in dedicated COVID-19 vaccine production facilities for supplying the annual COVID-19 booster vaccination, the severe healthcare impact of other vaccine-preventable diseases could be minimised by reinstating the production scale of other vaccines and biopharmaceuticals. Using the RNA and AVV platforms, booster COVID-19 vaccines may be produced relatively quickly, as may the production of new vaccines against emerging variants (which would still require clinical trials). A feasible option would be to combine the annual COVID-19 booster dose with the annual influenza vaccine into a multivalent vaccine^58^. For instance, it was found that the concomitant COVID-19 vaccination (ChAdOx1 or BNT162b2) with seasonal influenza vaccines raises no safety concerns and preserves the immune response to both vaccines in adults^58,59^. The UK Joint Committee on Vaccination and Immunisation recently advised that the flu vaccine may be co-administered with a booster or third dose of a COVID-19 vaccine^60^. In the longer term a multivalent COVID-19—influenza vaccine could also be developed. Although manufacturing multivalent vaccines comes with similar complexities and costs as manufacturing several monovalent vaccines, this would allow for the influenza vaccine itself to be produced on demand for the strain in circulation, without the need of forecasting the 3–4 most prevalent influenza strains more than 6 months ahead of the vaccination campaign^61,62^.

How financially viable maintaining surge vaccine manufacturing capacity during non-pandemic times is, depends on the ratio between the fixed and variable costs of a vaccine technology. Fixed costs, such as facility-dependent costs and labour, dominate AVV vaccine manufacturing as well as other cell-based vaccines and biopharmaceuticals (cf. Supplementary Fig. 3). By contrast, mRNA and saRNA vaccine manufacturing is driven by variable costs, such as raw material procurement. Therefore, maintaining surge capacity outside of epidemic or pandemic outbreaks would be more cost-effective for mRNA vaccines (and saRNA vaccines, once approved) compared to AVV vaccines. Surge capacity could be created by oversizing production facilities, with a view to ramping up production in case of an outbreak and speeding up both the development of relevant vaccines and their cGMP manufacturing.

In conclusion, the performance of the AVV, mRNA and saRNA vaccine platforms has been assessed in the context of COVID-19 pandemic response using techno-economic modelling and variance-based global sensitivity analysis. Both AVV and mRNA COVID-19 vaccines have been approved by regulatory authorities, while saRNA vaccines remain under clinical evaluation. The impact of key uncertain factors on selected KPIs, including productivity and resource-intensity indicators, has been quantified for all three vaccine production processes. Variations in the predicted annual productivity and cost per dose of AVV vaccines could be attributed primarily to uncertainty in the scale and titre/yield of the production process, and variations in the annual productivity and cost per dose of mRNA and saRNA vaccines primarily to uncertainty in the RNA amount per dose. Regarding productivity, the saRNA platform is predicted to be the fastest at producing 1 billion doses of COVID-19 vaccine or meeting the global vaccine demand, followed by the mRNA platform, and finally the AVV platform, by a substantial extent. The results of this assessment are sensitive to process specifics, such as DS amount per dose, batch lead time and production scale, some of which differ enourmously across the three platform technologies. The performance of the AVV platform could improve upon increasing the yield in the production bioreactor, while decreasing the RNA amount per dose could improve the production rates and volumes of mRNA and saRNA vaccines. Increasing the (thermo)stability of RNA vaccines could furthermore improve their deployability across the globe^47^.

The model-based assessment herein predicts that investments ranging from hundreds of millions to a few billion USD would be necessary to meet the current global projected vaccine shortfall and annual booster vaccination production. Albeit substantial, these investments remain marginal in regard to the mortality, healthcare and economic cost of the COVID-19 pandemic, estimated at over 10 trillion USD^51^. Overall, such model-based assessments could inform policymakers and vaccine manufacturers on the level of risk and on how to improve manufacturing and asset utilisation against COVID-19 and its variants. Finally, deployment of platform technologies dedicated to COVID-19 vaccine production could prevent a reduction in manufacturing throughput of other, non-COVID-19 vaccines and therapeutics, while enabling rapid-response vaccine development and production against a future epidemic or pandemic outbreak.

## Methods

### Vaccine production process modelling

The modelling of AVV, mRNA and saRNA DS production as well as drug product fill-to-finish was carried out using SuperPro Designer (Version 11, Build 2) by Intelligen, Inc. Further details are available in the Supplementary Information document.

### Data sources and assumptions

Information regarding mRNA and saRNA vaccine production processes and costs was obtained from the scientific literature^63–70^, patent databases^45,46,71–73^, from cGMP grade material suppliers^74,75^ and from experts^76–78^. Information regarding mRNA DS amount per dose was based on clinical trial databases^79–82^ and the scientific literature^83^. For saRNA vaccines the DS amount per dose was obtained from the clinical trial registry^84^. Information on AVV vaccine production was obtained from the scientific literature^85–88^. The AVV vaccine production process was modelled based on the manufacturing of the replication-deficient chimpanzee adenovirus-vectored (ChAdOx1) vaccine which was co-developed by Oxford University and AstraZeneca plc. Information on AVV DS amount per dose was based on clinical trial databases^89–95^. Similar process and cost modelling analyses of AVV and mRNA COVID-19 vaccine production processes have also been recently and independently published^96,97^. Information on fill-to-finish technologies was obtained from the literature^19,98–100^, equipment suppliers^18,20,101,102^ and industry experts^103,104^. Additional production process data for all DS and drug product manufacturing processes were retreived from the equipment, materials, utilities and cost databases in SuperPro Designer^105^. The annualised CapEx is included in the OpEx. The CapEx value is also presented individually, in order to illustrate the financial requirements for building new facilities.

### Sensitivity analysis

Variance-based global sensitivity analyses were conducted using SobolGSA Version 3.1.1^106^ under MatLab R2020a. 10,000 quasi-random scenarios were generated using Sobol sequences^27–29,107^ according to the process parameter ranges and distributions in Table 1 then passed to SuperPro Designer for evaluating the techno-economic KPIs in each scenario. A metamodel was generated in SobolGSA using the random-sampling high dimensional model representation^108,109^ based on which the main-effect and total-effect Sobol indices were derived^24^. A further 1250 uncertainty scenarios were simulated in SuperPro Designer to test the predictions of the metamodel. The link between SobolGSA and SuperPro Designer was enabled by a Component Object Model interface in MS-Excel VBA available from MS-Office 365 Enterprise. Further details are available in the Supplementary Information document. Data processing and visualisation/plotting is also described in the Supplementary Information document.

### Reporting summary

Further information on research design is available in the Nature Research Reporting Summary linked to this article.

## Supplementary information

Supplementary Information Document

REPORTING SUMMARY

## Data Availability

Data is available from: https://github.com/ZKis-ZK/RNA_AVV_vaccine_production-cost_modelling_global_sensitivity_analysis.

## Code availability

The code and scripts are available from: https://github.com/ZKis-ZK/RNA_AVV_vaccine_production-cost_modelling_global_sensitivity_analysis.
