# Supplementary material for: Pandemic-response adenoviral vector and RNA vaccine manufacturing
Source: NPJ Vaccines. 2022 Mar 2;7:29. doi: 10.1038/s41541-022-00447-3 (PMC8891260; doi:10.1038/s41541-022-00447-3)
Supplement: Supplementary file 2 — REPORTING SUMMARY [file 41541_2022_447_MOESM2_ESM.pdf]

nature portfolio

Corresponding author(s): Zoltan Kis

Last updated by author(s): Jan 10, 2022

Reporting Summary

Nature Portfolio wishes to improve the reproducibility of the work that we publish. This form provides structure for consistency and transparency in reporting. For further information on Nature Portfolio policies, see our Editorial Policies and the Editorial Policy Checklist.

Statistics

For all statistical analyses, confirm that the following items are present in the figure legend, table legend, main text, or Methods section.

n/a	Confirmed	
☐	☒	The exact sample size (*n*) for each experimental group/condition, given as a discrete number and unit of measurement
☐	☒	A statement on whether measurements were taken from distinct samples or whether the same sample was measured repeatedly
☒	☐	The statistical test(s) used AND whether they are one- or two-sided<br>*Only common tests should be described solely by name; describe more complex techniques in the Methods section.*
☒	☐	A description of all covariates tested
☐	☒	A description of any assumptions or corrections, such as tests of normality and adjustment for multiple comparisons
☐	☒	A full description of the statistical parameters including central tendency (e.g. means) or other basic estimates (e.g. regression coefficient) AND variation (e.g. standard deviation) or associated estimates of uncertainty (e.g. confidence intervals)
☒	☐	For null hypothesis testing, the test statistic (e.g. F, t, r) with confidence intervals, effect sizes, degrees of freedom and P value noted<br>*Give P values as exact values whenever suitable.*
☐	☒	For Bayesian analysis, information on the choice of priors and Markov chain Monte Carlo settings
☒	☐	For hierarchical and complex designs, identification of the appropriate level for tests and full reporting of outcomes
☒	☐	Estimates of effect sizes (e.g. Cohen's *d*, Pearson's *r*), indicating how they were calculated

Our web collection on statistics for biologists contains articles on many of the points above.

Software and code

Policy information about availability of computer code

Data collection	Information regarding mRNA and saRNA vaccine production processes and costs was obtained from the scientific literature [44–51], patent databases [30–33,43], from cGMP grade material suppliers [97,98] and from experts [99–101]. Information regarding mRNA drug substance amount per dose was based on clinical trial databases [61,62,102,103] and the scientific literature [104]. For saRNA vaccines the drug substance amount per dose was obtained from the clinical trial registry [105]. Information on AVV vaccine production was obtained from the scientific literature [39–42]. The AVV vaccine production process was modelled based on the manufacturing of the replication-deficient chimpanzee adenovirus-vectored (ChAdOx1) vaccine which was co-developed by Oxford University and AstraZeneca plc. Information on AVV drug substance amount per dose was based on clinical trial databases [59,60, 63,64,106–108]. Similar process and cost modelling analyses of AVV and mRNA COVID-19 vaccine production processes have also been recently and independently published [109,110]. Information on fill-to-finish technologies was obtained from the literature [19,111–113], equipment suppliers [18, 20,114,115] and industry experts [116,117]. Additional production process data for all drug substance and drug product manufacturing processes were retreived from the equipment, materials, utilities and cost databases in SuperPro Designer [118]. The annualised CapEx is included in the OpEx. The CapEx value is also presented individually, in order to illustrate the financial requirements for building new facilities.
Data analysis	SuperPro Designer (Version 11, Build 2) by Intelligen, Inc.<br>SobolGSA Version 3.1.1

For manuscripts utilizing custom algorithms or software that are central to the research but not yet described in published literature, software must be made available to editors and reviewers. We strongly encourage code deposition in a community repository (e.g. GitHub). See the Nature Portfolio guidelines for submitting code & software for further information.

Data

Policy information about availability of data

All manuscripts must include a data availability statement. This statement should provide the following information, where applicable:

- Accession codes, unique identifiers, or web links for publicly available datasets
- A description of any restrictions on data availability
- For clinical datasets or third party data, please ensure that the statement adheres to our policy

Data is available from: https://github.com/ZKis-ZK/RNA_AVV_vaccine_production-cost_modelling_global_sensitivity_analysis

Field-specific reporting

Please select the one below that is the best fit for your research. If you are not sure, read the appropriate sections before making your selection.

☒ Life sciences ☐ Behavioural & social sciences ☐ Ecological, evolutionary & environmental sciences

For a reference copy of the document with all sections, see nature.com/documents/nr-reporting-summary-flat.pdf

Life sciences study design

All studies must disclose on these points even when the disclosure is negative.

Sample size	10k simulation runs:<br>Variance-based global sensitivity analyses were conducted using SobolGSA Version 3.1.1 [119] under MatLab R2020a. 10,000 quasi-random scenarios were generated using Sobol sequences [27–29,120] according to the process parameter ranges and distributions in Table 1 then passed to SuperPro Designer for evaluating the techno-economic KPIs in each scenario. A metamodel was generated in SobolGSA using the random-sampling high dimensional model representation (RS-HDMR) [121,122] based on which the main-effect and total-effect Sobol indices were derived [24]. A further 1,250 uncertainty scenarios were simulated in SuperPro Designer to test the predictions of the metamodel. The link between SobolGSA and SuperPro Designer was enabled by a Component Object Model (COM) interface in MS-Excel VBA available from from MS-Office 365 Enterprise. Further details are available in the SI document. Data processing and visualisation/plotting is also described in the SI document.
Data exclusions	All the simulation data is available in the SI file
Replication	The simulation runs were replicated over 5 times, in each run over 10k simulations were performed
Randomization	quasi-random sampling based on Sobol sequences
Blinding	Not applicable

Reporting for specific materials, systems and methods

We require information from authors about some types of materials, experimental systems and methods used in many studies. Here, indicate whether each material, system or method listed is relevant to your study. If you are not sure if a list item applies to your research, read the appropriate section before selecting a response.

Materials & experimental systems

n/a	Involved in the study
☒	☐ Antibodies
☒	☐ Eukaryotic cell lines
☒	☐ Palaeontology and archaeology
☒	☐ Animals and other organisms
☒	☐ Human research participants
☒	☐ Clinical data
☒	☐ Dual use research of concern

Methods

n/a	Involved in the study
☒	☐ ChIP-seq
☒	☐ Flow cytometry
☒	☐ MRI-based neuroimaging
